# Effects of exogenous ketone supplementation on blood ketone, glucose, triglyceride, and lipoprotein levels in Sprague–Dawley rats

**DOI:** 10.1186/s12986-016-0069-y

**Published:** 2016-02-04

**Authors:** Shannon L. Kesl, Angela M. Poff, Nathan P. Ward, Tina N. Fiorelli, Csilla Ari, Ashley J. Van Putten, Jacob W. Sherwood, Patrick Arnold, Dominic P. D’Agostino

**Affiliations:** Department of Molecular Pharmacology and Physiology, Morsani College of Medicine, University of South Florida, 12901 Bruce B. Downs Blvd. MDC8, Tampa, FL 33612 USA; Savind Inc, 205 South Main Street, Seymore, IL 61875 USA

**Keywords:** Ketogenic diet, Ketone ester, Ketone supplement, Appetite, β-hydroxybutyrate, Hyperketonemia, Triglycerides

## Abstract

**Background:**

Nutritional ketosis induced by the ketogenic diet (KD) has therapeutic applications for many disease states. We hypothesized that oral administration of exogenous ketone supplements could produce sustained nutritional ketosis (>0.5 mM) without carbohydrate restriction.

**Methods:**

We tested the effects of 28-day administration of five ketone supplements on blood glucose, ketones, and lipids in male Sprague–Dawley rats. The supplements included: 1,3-butanediol (BD), a sodium/potassium β-hydroxybutyrate (βHB) mineral salt (BMS), medium chain triglyceride oil (MCT), BMS + MCT 1:1 mixture, and 1,3 butanediol acetoacetate diester (KE). Rats received a daily 5–10 g/kg dose of their respective ketone supplement via intragastric gavage during treatment. Weekly whole blood samples were taken for analysis of glucose and βHB at baseline and, 0.5, 1, 4, 8, and 12 h post-gavage, or until βHB returned to baseline. At 28 days, triglycerides, total cholesterol and high-density lipoprotein (HDL) were measured.

**Results:**

Exogenous ketone supplementation caused a rapid and sustained elevation of βHB, reduction of glucose, and little change to lipid biomarkers compared to control animals.

**Conclusions:**

This study demonstrates the efficacy and tolerability of oral exogenous ketone supplementation in inducing nutritional ketosis independent of dietary restriction.

## Background

Emerging evidence supports the therapeutic potential of the ketogenic diet (KD) for a variety of disease states, leading investigators to research methods of harnessing the benefits of nutritional ketosis without the dietary restrictions. The KD has been used as an effective non-pharmacological therapy for pediatric intractable seizures since the 1920s [1–3]. In addition to epilepsy, the ketogenic diet has elicited significant therapeutic effects for weight loss and type-2 diabetes (T2D) [4]. Several studies have shown significant weight loss on a high fat, low carbohydrate diet without significant elevations of serum cholesterol [5–12]. Another study demonstrated the safety and benefits of long-term application of the KD in T2D patients. Patients exhibited significant weight loss, reduction of blood glucose, and improvement of lipid markers after eating a well-formulated KD for 56 weeks [13]. Recently, researchers have begun to investigate the use of the KD as a treatment for acne, polycystic ovary syndrome (PCOS), cancer, amyotrophic lateral sclerosis (ALS), traumatic brain injury (TBI) and Alzheimer’s disease (AD) with promising preliminary results [14–26].

The classical KD consists of a 4:1 ratio of fat to protein and carbohydrate, with 80–90 % of total calories derived from fat [27]. The macronutrient ratio of the KD induces a metabolic shift towards fatty acid oxidation and hepatic ketogenesis, elevating the ketone bodies acetoacetate (AcAc) and β-hydroxybutyrate (βHB) in the blood. Acetone, generated by decarboxylation of AcAc, has been shown to have anticonvulsant properties [28–32]. Ketone bodies are naturally elevated to serve as alternative metabolic substrates for extra-hepatic tissues during the prolonged reduction of glucose availability, suppression of insulin, and depletion of liver glycogen, such as occurs during starvation, fasting, vigorous exercise, calorie restriction, or the KD. Although the KD has clear therapeutic potential, several factors limit the efficacy and utility of this metabolic therapy for widespread clinical use. Patient compliance to the KD can be low due to the severe dietary restriction - the diet being generally perceived as unpalatable - and intolerance to high-fat ingestion. Maintaining ketosis can be difficult as consumption of even a small quantity of carbohydrates or excess protein can rapidly inhibit ketogenesis [33, 34]. Furthermore, enhanced ketone body production and tissue utilization by the tissues can take several weeks (keto-adaptation), and patients may experience mild hypoglycemic symptoms during this transitional period [35].

Recent studies suggest that many of the benefits of the KD are due to the effects of ketone body metabolism. Interestingly, in studies on T2D patients, improved glycemic control, improved lipid markers, and retraction of insulin and other medications occurred before weight loss became significant. Both βHB and AcAc have been shown to decrease mitochondrial reactive oxygen species (ROS) production [36–39]. Veech et al. have summarized the potential therapeutic uses for ketone bodies [28, 40]. They have demonstrated that exogenous ketones favorably alter mitochondrial bioenergetics to reduce the mitochondrial NAD couple, oxidize the co-enzyme Q, and increase the ΔG’ (free enthalpy) of ATP hydrolysis [41]. Ketone bodies have been shown to increase the hydraulic efficiency of the heart by 28 %, simultaneously decreasing oxygen consumption while increasing ATP production [42]. Thus, elevated ketone bodies increase metabolic efficiency and as a consequence, reduce superoxide production and increase reduced glutathione [28]. Sullivan et al. demonstrated that mice fed a KD for 10–12 days showed increased hippocampal uncoupling proteins, indicative of decreased mitochondrial-produced ROS [43]. Bough et al. showed an increase of mitochondrial biogenesis in rats maintained on a KD for 4–6 weeks [44, 45]. Recently, Shimazu et al. reported that βHB is an exogenous and specific inhibitor of class I histone deacetylases (HDACs), which confers protection against oxidative stress [38]. Ketone bodies have also been shown to suppress inflammation by decreasing the inflammatory markers TNF-a, IL-6, IL-8, MCP-1, E-selectin, I-CAM, and PAI-1 [8, 46, 47]. Therefore, it is thought that ketone bodies themselves confer many of the benefits associated with the KD.

Considering both the broad therapeutic potential and limitations of the KD, an oral exogenous ketone supplement capable of inducing sustained therapeutic ketosis without the need for dietary restriction would serve as a practical alternative. Several natural and synthetic ketone supplements capable of inducing nutritional ketosis have been identified. Desrochers et al. elevated ketone bodies in the blood of pigs (>0.5 mM) using exogenous ketone supplements: (R, S)-1,3 butanediol and (R, S)-1,3 butanediol-acetoacetate monoesters and diester [48]. In 2012, Clarke et al. demonstrated the safety and efficacy of chronic oral administration of a ketone monoester of R-βHB in rats and humans [49, 50]. Subjects maintained elevated blood ketones without dietary restriction and experienced little to no adverse side effects, demonstrating the potential to circumvent the restrictive diet typically needed to achieve therapeutic ketosis. We hypothesized that exogenous ketone supplements could produce sustained hyperketonemia (>0.5 mM) without dietary restriction and without negatively influencing metabolic biomarkers, such as blood glucose, total cholesterol, HDL, LDL, and triglycerides. Thus, we measured these biomarkers during a 28-day administration of the following ketone supplements in rats: naturally-derived ketogenic supplements included medium chain triglyceride oil (MCT), sodium/potassium -βHB mineral salt (BMS), and sodium/potassium -βHB mineral salt + medium chain triglyceride oil 1:1 mixture (BMS + MCT) and synthetically produced ketogenic supplements included 1, 3-butanediol (BD), 1, 3-butanediol acetoacetate diester/ ketone ester (KE).

## Methods

### Synthesis and formulation of ketone supplements

KE was synthesized as previously described [29]. BMS is a novel agent (sodium/potassium- βHB mineral salt) supplied as a 50 % solution containing approximately 375 mg/g of pure βHB and 125 mg/g of sodium/potassium. Both KE and BMS were developed and synthesized in collaboration with Savind Inc. Pharmaceutical grade MCT oil (~65 % caprylic triglyceride; 45 % capric triglyceride) was purchased from Now Foods (Bloomingdale, IL). BMS was formulated in a 1:1 ratio with MCT at the University of South Florida (USF), yielding a final mixture of 25 % water, 25 % pure βHB mineral salt and 50 % MCT. BD was purchased from Sigma-Aldrich (Prod # B84785, Milwaukee, WI).

### Daily gavage to induce dietary ketosis

Animal procedures were performed in accordance with the University of South Florida Institutional Animal Care and Use Committee (IACUC) guidelines (Protocol #0006R). Juvenile male Sprague–Dawley rats (275–325 g, Harlan Laboratories) were randomly assigned to one of six study groups: control (water, *n* = 11), BD (*n* = 11), KE (*n* = 11), MCT (*n* = 10), BMS (*n* = 11), or BMS + MCT (*n* = 12). Caloric density of standard rodent chow and dose of ketone supplements are listed in Table 1. On days 1–14, rats received a 5 g/kg body weight dose of their respective treatments via intragastric gavage. Dosage was increased to 10 g/kg body weight for the second half of the study (days 15–28) for all groups except BD and KE to prevent excessive hyperketonemia (ketoacidosis). Each daily dose of BMS would equal ~1000–1500 mg of βHB, depending on the weight of the animal. Intragastric gavage was performed at the same time daily, and animals had *ad libitum* access to standard rodent chow 2018 (Harlan Teklad) for the duration of the study. The macronutrient ratio the standard rodent chow was 62.2, 23.8 and 14 % of carbohydrates, protein and fat respectively.

**Table 1:** Caloric density and dose of ketone supplements

Macronutrient Information	Standard Diet	Water	BMS + MCT	BMS	MCT	KE	BD
% Cal from Fat	18.0	0.0	50.0	N/A	100.0	N/A	N/A
% Cal from Protein	24.0	0.0	N/A	N/A	0.0	N/A	N/A
% Cal from Carbohydrates	58.0	0.0	N/A	N/A	0.0	N/A	N/A
Total Caloric Density (Kcal/g)	3.1	0.0	5.1	1.9	8.3	5.6	6.0
Dose 0–14 Days (g/kg)	*ad libitum*	N/A	5.0	5.0	5.0	5.0	5.0
Dose 15–28 Days (g/kg)	*ad libitum*	N/A	10.0	10.0	10.0	5.0	5.0

### Measurement and analysis of blood glucose, ketones, and lipids

Every 7 days, animals were briefly fasted (4 h, water available) prior to intragastric gavage to standardize levels of blood metabolites prior to glucose and βHB measurements at baseline. Baseline (time 0) was immediately prior to gavage. Whole blood samples (10 μL) were taken from the saphenous vein for analysis of glucose and βHB levels with the commercially available glucose and ketone monitoring system Precision Xtra™ (Abbott Laboratories, Abbott Park, IL). Blood glucose and βHB were measured at 0, 0.5, 1, 4, 8, and 12 h after test substance administration, or until βHB returned to baseline levels. Food was returned to animals after blood analysis at time 0 and gavage. At baseline and week 4, whole blood samples (10 μL) were taken from the saphenous vein immediately prior to gavage (time 0) for analysis of total cholesterol, high-density lipoprotein (HDL), and triglycerides with the commercially available CardioChek™ blood lipid analyzer (Polymer Technology Systems, Inc., Indianapolis, IN). Low-density lipoprotein (LDL) cholesterol was calculated from the three measured lipid levels using the Friedewald equation: (LDL Cholesterol = Total Cholesterol - HDL - (Triglycerides/5)) [51, 52]. Animals were weighed once per week to track changes in body weight associated with hyperketonemia.

### Organ weight and collection

On day 29, rats were sacrificed via deep isoflurane anesthesia, exsanguination by cardiac puncture, and decapitation 4–8 h after intragastric gavage, which correlated to the time range where the most significantly elevated blood βHB levels were observed. Brain, lungs, liver, kidneys, spleen and heart were harvested, weighed (AWS-1000 1 kg portable digital scale (AWS, Charleston, SC)), and flash-frozen in liquid nitrogen or preserved in 4 % paraformaldehyde for future analysis.

### Statistics

All data are presented as the mean ± standard deviation (SD). Data analysis was performed using GraphPad PRISM™ version 6.0a and IBM SPSS Statistics 22.0. Results were considered significant when p < 0.05. Triglyceride and lipoprotein profile data were analyzed using One-Way ANOVA. Blood ketone and blood glucose were compared to control at the applicable time points using a Two-Way ANOVA. Correlation between blood βHB and glucose levels in ketone supplemented rats was compared to controls using ANCOVA analysis. Organ and body weights were analyzed using One-Way ANOVA. Basal blood ketone and blood glucose levels were analyzed using Two-Way ANOVA. All mean comparisons were carried out using Tukey’s multiple comparisons post-hoc test.

## Results

### Effect of ketone supplementation on triglycerides and lipoproteins

**Fig. 1:**
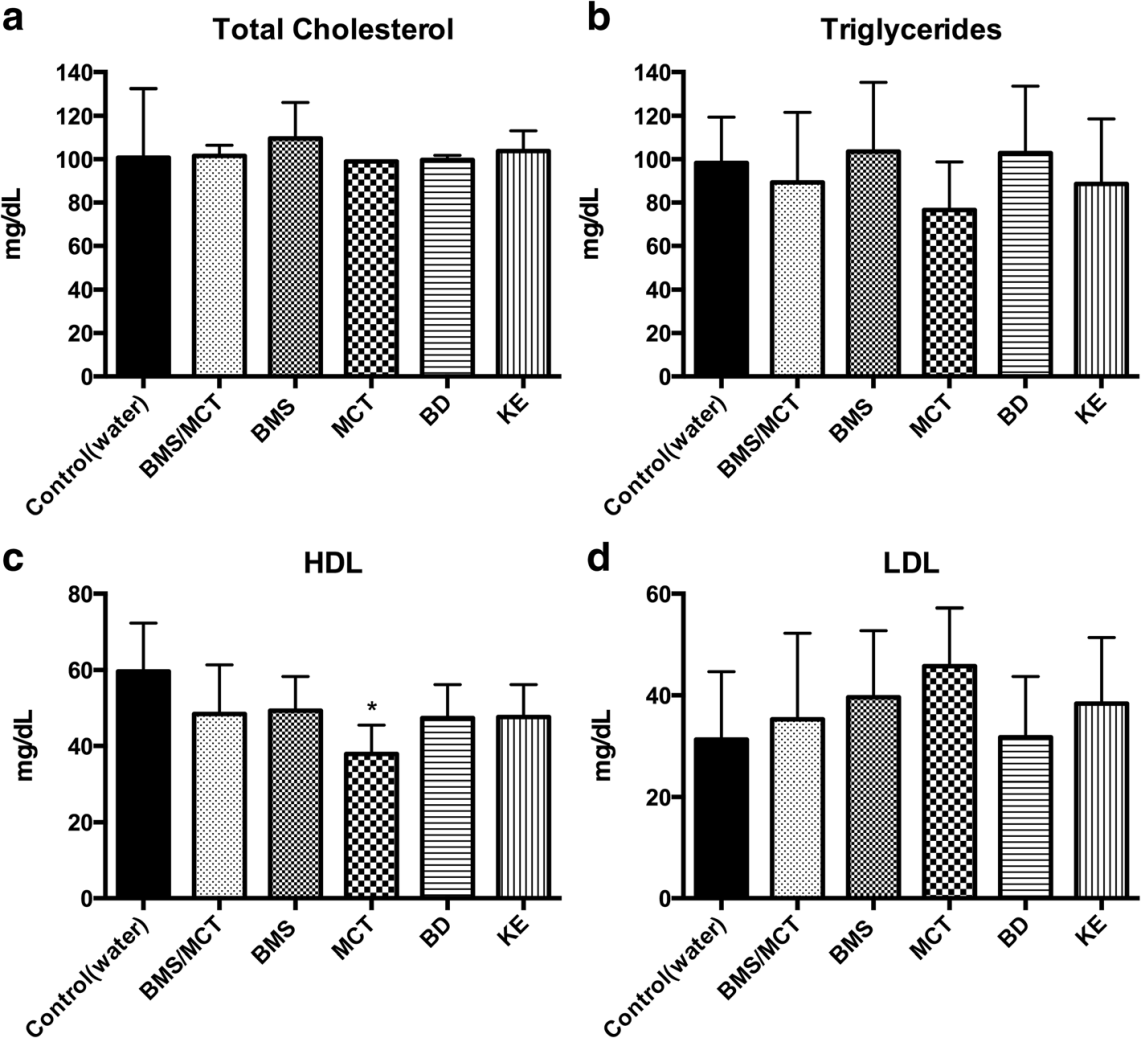
Effects of ketone supplementation on triglycerides and lipoproteins: Ketone supplementation causes little change in triglycerides and lipoproteins over a 4-week study. Graphs show concentrations at 4-weeks of total cholesterol (**a**), Triglycerides (**b**), LDL (**c**), and HDL (**d**). MCT supplemented rats had signfiicantly reduced concentration of HDL blood levels compared to control (*p* < 0.001) (**b**). One-Way ANOVA with Tukey’s post hoc test, results considered significant if *p* < 0.05. Error bars represent mean (SD)

Baseline measurements showed no significant changes in triglycerides or the lipoproteins (data not shown). Data represent triglyceride and lipoprotein concentrations measured after 4 weeks of daily exogenous ketone supplementation. No significant change in total cholesterol was observed at 4 weeks for any of the ketone treatment groups compared to control. (Fig. 1a). No significant difference was detected in triglycerides for any ketone supplement compared to control (Fig. 1b). MCT supplemented animals had a significant reduction in HDL blood levels compared to control (p < 0.001) (Fig. 1c). LDL levels in ketone-supplemented animals did not significantly differ from controls (Fig. 1d).

### Ketone supplementation causes rapid and sustained elevation of βHB

**Fig. 2:**
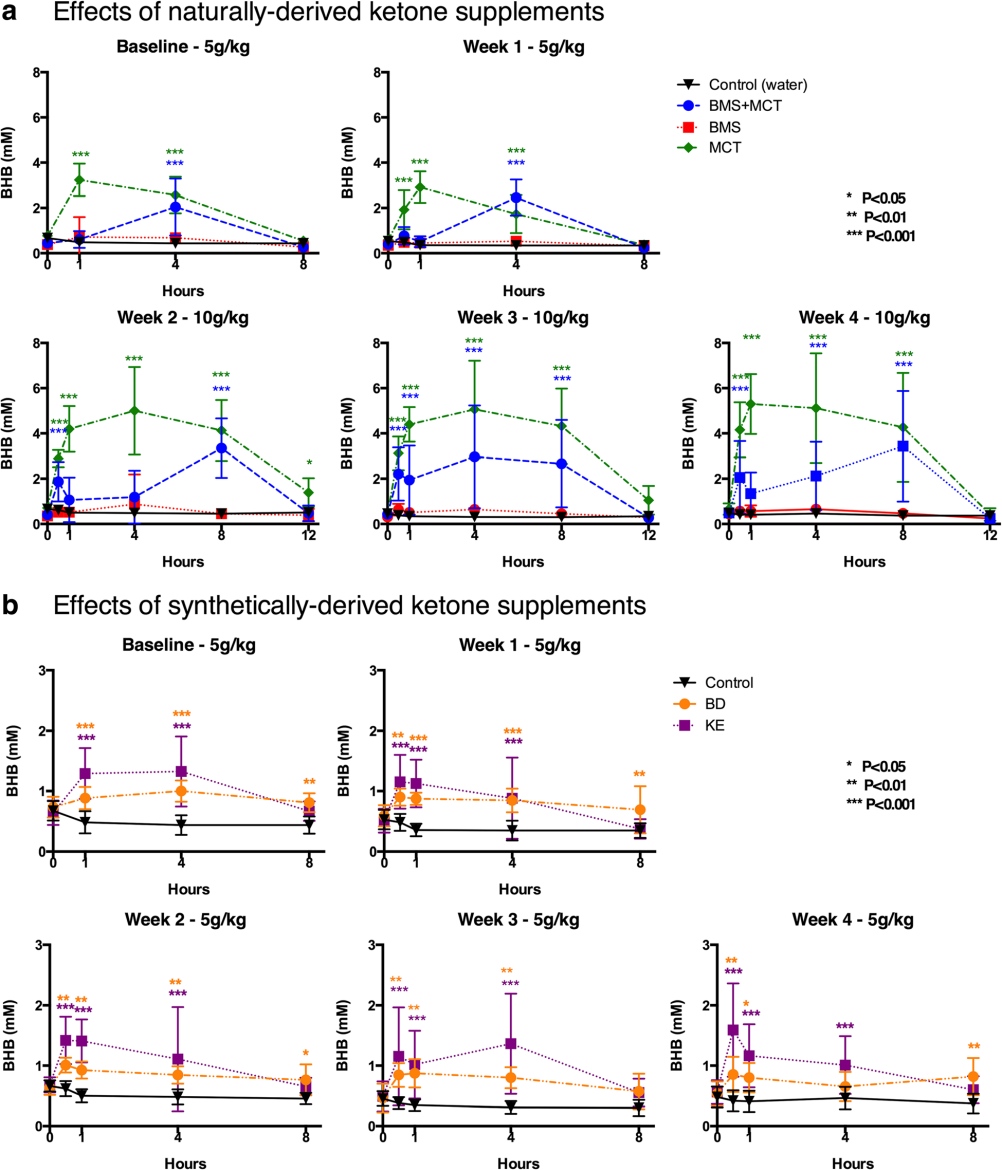
Effects of ketone supplementation on blood βHB. **a**, **b** Blood βHB levels at times 0, 0.5, 1, 4, 8, and 12 h post intragastric gavage for ketone supplements tested. **a** BMS + MCT and MCT supplementation rapidly elevated and sustained significant βHB elevation compared to controls for the duration of the 4-week dose escalation study. BMS did not significantly elevate βHB at any time point tested compared to controls. **b** BD and KE supplements, maintained at 5 g/kg, significantly elevated βHB levels for the duration of the 4-week study. Two-Way ANOVA with Tukey’s post hoc test, results considered significant if *p* < 0.05. Error bars represent mean (SD)

Over the 28-day experiment, ketone supplements administered daily significantly elevated blood ketone levels without dietary restriction (Fig. 2a, b). Naturally derived ketogenic supplements including MCT (5 g/kg) elicited a significant rapid elevation in blood βHB within 30–60 min that was sustained for 8 h. BMS + MCT (5 g/kg) elicited a significant elevation in blood βHB at 4 h, which was no longer significant at 8 h. BMS (5 g/kg) did not elicit a significant elevation in blood βHB at any time point. For days 14–28, BMS + MCT (10 g/kg) and MCT (10 g/kg) elevated blood βHB levels within 30 min and remained significantly elevated for up to 12 h. We observed a delay in the peak elevation of blood βHB: BMS + MCT peaked at 8 h instead of at 4 h and MCT at 4 h instead of at 1 h. Blood βHB levels in the BMS group did not show significant elevation at any time point, even after dose escalation (Fig. 2a). Synthetically derived ketogenic supplements including KE and BD supplementation rapidly elevated blood βHB within 30 min and was sustained for 8 h. For the rats receiving ketone supplementation in the form of BD or the KE, dosage was kept at 5 g/kg to prevent adverse effects associated with hyperketonemia. The Precision Xtra™ ketone monitoring system measures βHB only; therefore, total blood ketone levels (βHB + AcAc) would be higher than measured. For each of these groups, the blood βHB profile remained consistent following daily ketone supplementation administration over the 4-week duration. (Fig. 2b).

### Ketone supplementation causes a significant decrease of blood glucose

**Fig. 3:**
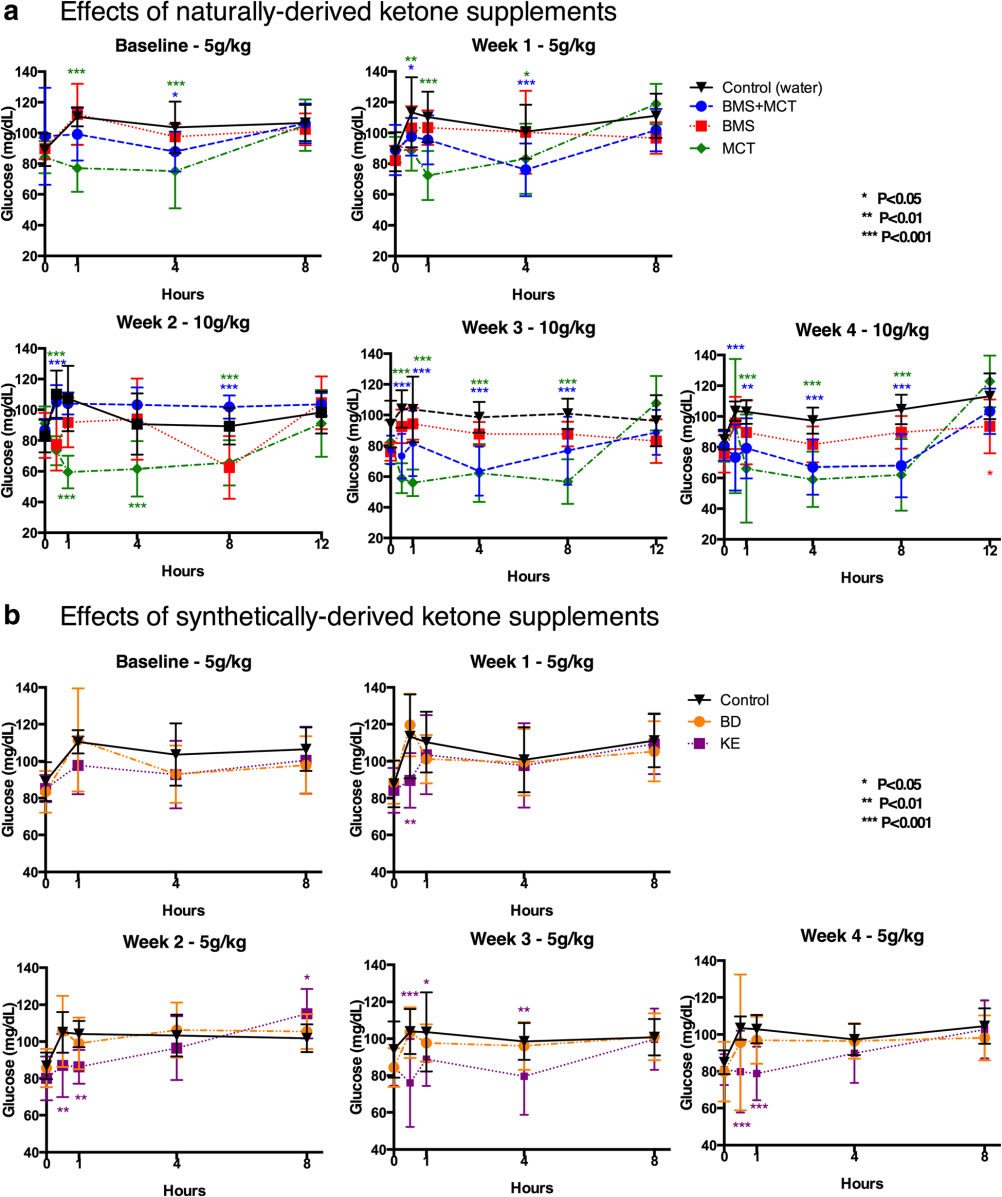
Effects of ketone supplementation on blood glucose. **a**, **b** Blood glucose levels at times 0, 0.5, 1, 4, 8, and 12 h (for 10 dose) post intragastric gavage for ketone supplements tested. **a** Ketone supplements BMS + MCT and MCT significantly reduced blood glucose levels compared to controls for the duration of the 4-week study. BMS significantly lowered blood glucose only at 8 h/week 1 and 12 h/week 3 (**b**) KE, maintained at 5 g/kg, significantly reduced blood glucose compared to controls from week 1–4. BD did not significantly affect blood glucose levels at any time point during the 4-week study. Two-Way ANOVA with Tukey’s post hoc test, results considered significant if *p* < 0.05. Error bars represent mean (SD)

Administration of ketone supplementation significantly reduced blood glucose over the course of the study (Fig. 3a, b). MCT (5 g/kg) decreased blood glucose compared to control within 30 min which was sustained for 8 h at baseline and at week 1. MCT (10 g/kg) likewise decreased blood glucose within 30 min and lasted through the 12 h time point during weeks 2, 3, and 4. BMS + MCT (5 g/kg) lowered blood glucose compared to control from hours 1–8 only at week 1. BMS + MCT (10 g/kg) lowered blood glucose compared to control within 30 min and remained low through the 12 h time point at weeks 2, 3, and 4. Rats supplemented with BMS had lower blood glucose compared to control at 12 h in week 4 (10) (Fig. 3a). Administration of BD did not significantly change blood glucose levels at any time point during the 4-week study. KE (5 g/kg) significantly lowered blood glucose levels at 30 min for week 1, 2, 3, and 4 and was sustained through 1 h at weeks 2–4 and sustained to 4 h at week 3. (Fig. 3b).

### Hyperketonemia suppresses blood glucose levels

**Fig. 4:**
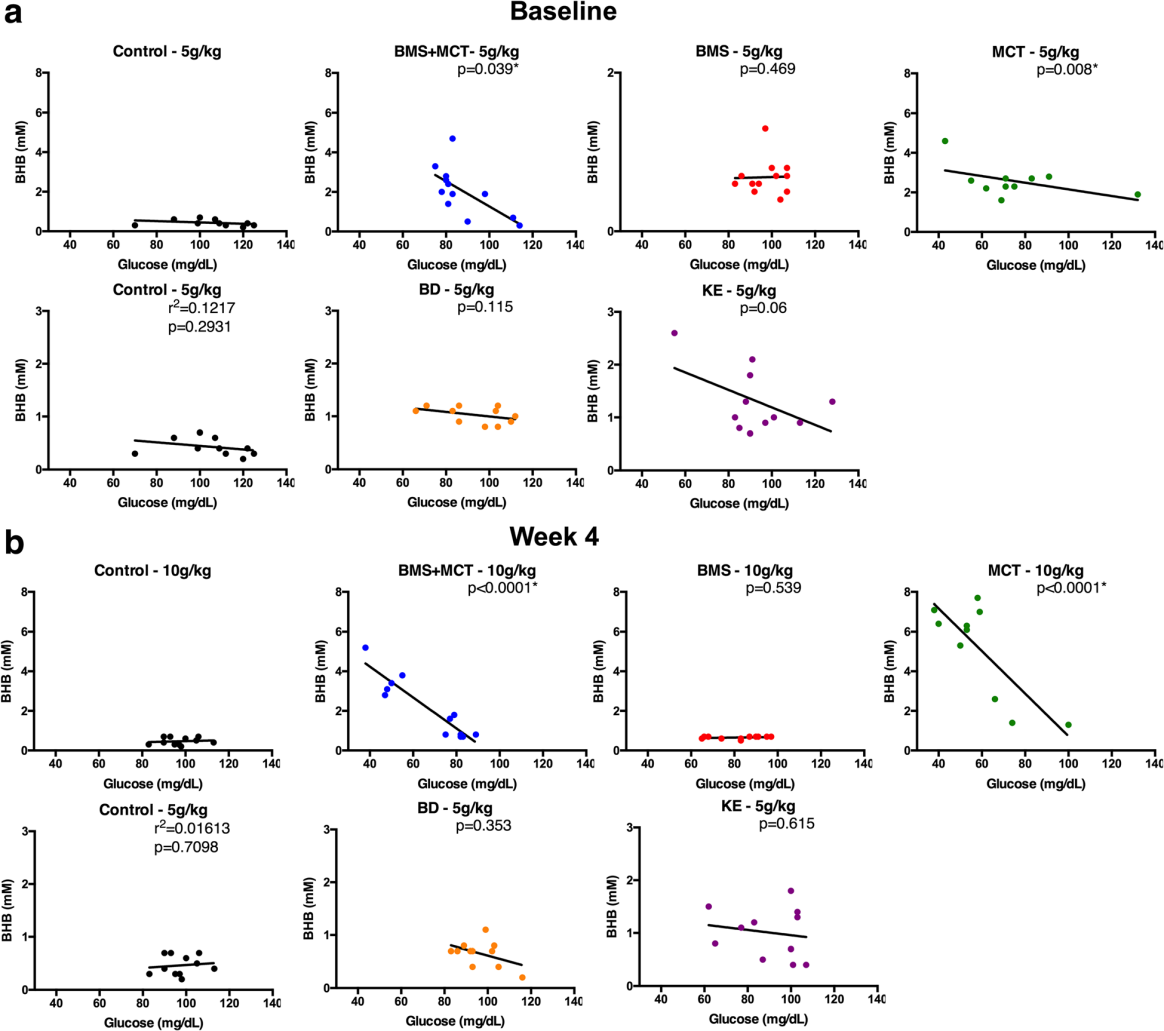
Relationship between blood ketone and glucose levels: **a** BMS + MCT (5 g/kg) supplemented rats demonstrated a significant inverse relationship between elevated blood ketone levels and decreased blood ketone levels (r^2^ = 0.4314, p = 0.0203). **b** At week 4, BMS + MCT (10 g/kg) and MCT (10 g/kg) showed a significant correlation between blood ketone levels and blood glucose levels (r2 = 0.8619, *p* < 0.0001; r^2^ = 0.6365, p = 0.0057). Linear regression analysis, results considered significant if *p* < 0.05

At baseline, 4 h after intragastric gavage, the elevation of blood ketones was inversely related to the reduction of blood glucose compared to controls following the administration of MCT (5 g/kg) (p = 0.008) and BMS + MCT (5 g/kg) (p = 0.039) . There was no significant correlation between blood ketone levels and blood glucose levels compared to controls for any other ketone supplemented group at baseline (Fig. 4a). At week 4, 4 h after intragastric gavage, there was a significant correlation between blood ketone levels and blood glucose levels compared to controls in MCT (10 g/kg) and BMS + MCT (10 g/kg) (*p* < 0.0001, *p* < 0.0001) (Fig. 4b).

### Ketone supplementation changes organ weight and decreases body weight

**Fig. 5:**
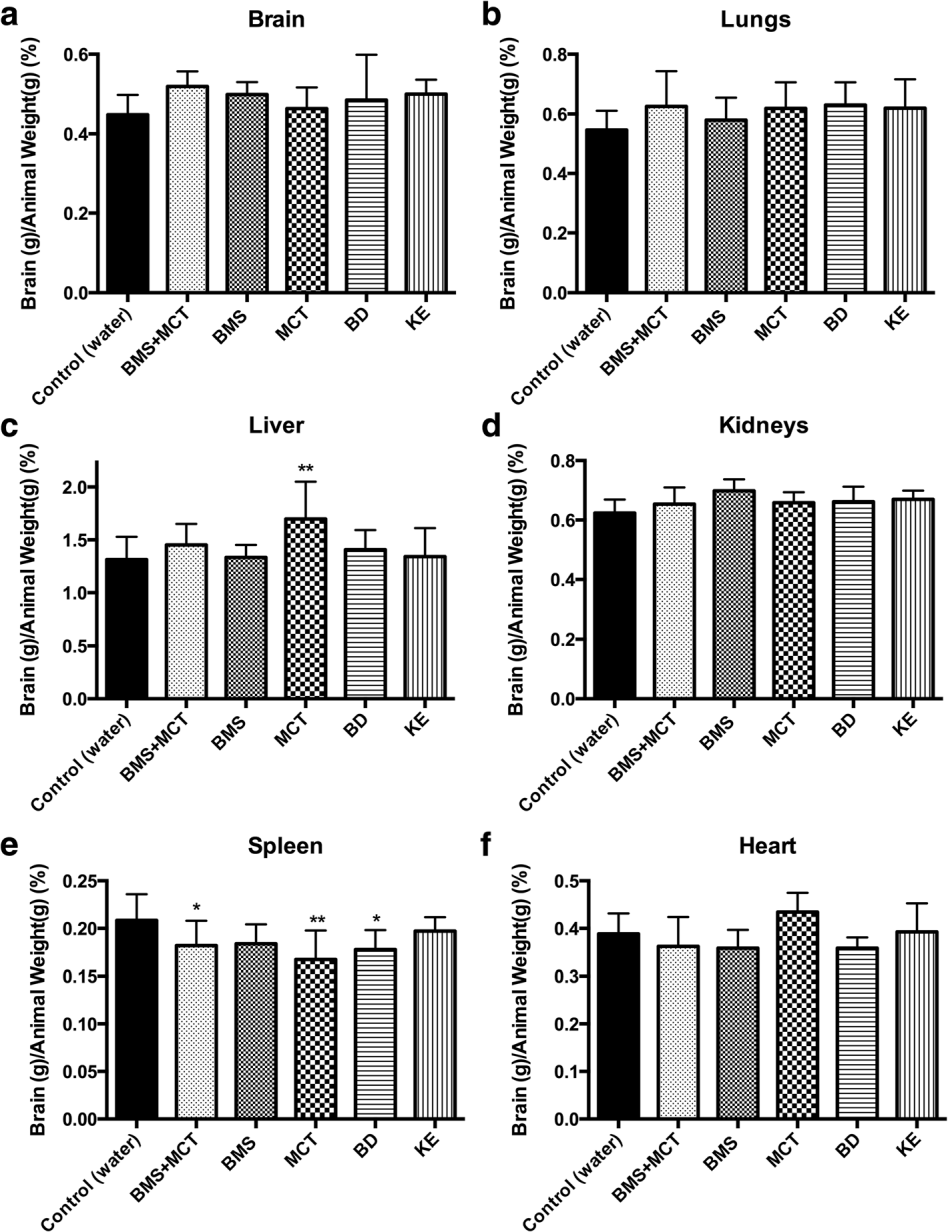
Effects of ketone supplementation on organ weight: Data is represented as a percentage of organ weight to body weight. **a**, **b**, **d**, **f** Ketone supplements did not significantly affect the weight of the brain, lungs, kidneys or heart. **c** Liver weight was significantly increased as compared to body weight in response to administered MCT ketone supplement compared to control at the end of the study (day 29) (*p* < 0.001). **e** Rats supplemented with BMS + MCT, MCT, and BD had significantly smaller spleen percentage as compared to controls (*p* < 0.05, *p* < 0.001, *p* < 0.05). Two-Way ANOVA with Tukey’s post-hoc test; results considered significant if *p* < 0.05. Error bars represent mean (SD)

**Fig. 6:**
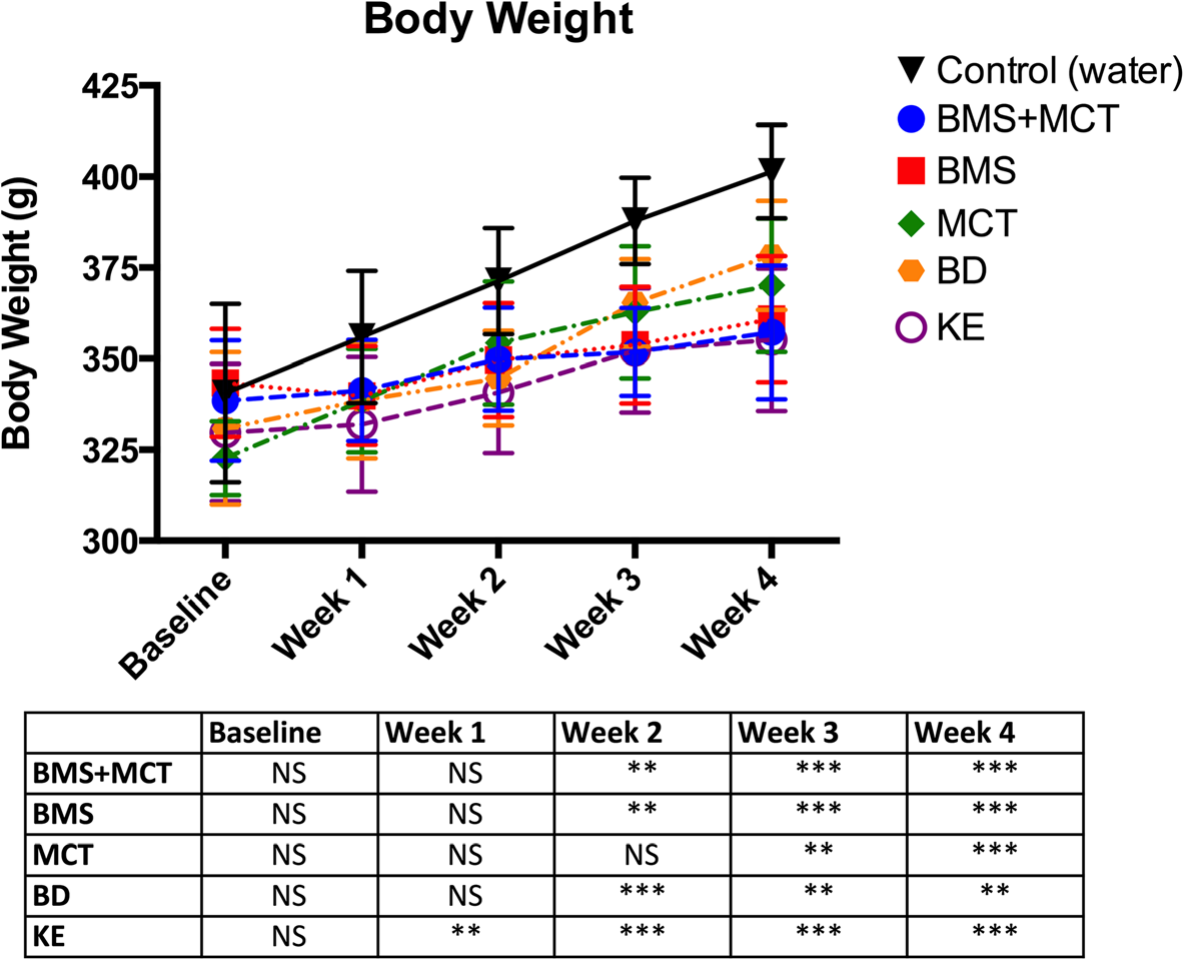
Effects of ketone supplementation on body weight: Rats administered ketone supplements gained less weight over the 4-week period; however, did not lose weight and maintained healthy range for age. KE supplemented rats gained significantly less weight during the entire 4-week study compared to controls. BMS + MCT, BMS, and BD supplemented rats gained significantly less weight than controls over weeks 2–4.MCT supplemented rats gained significantly less weight than controls over weeks 3–4, Two-Way ANOVA with Tukey’s post hoc test, results considered significant if *p* < 0.05. Error bars represent mean (SD)

**Fig. 7:**
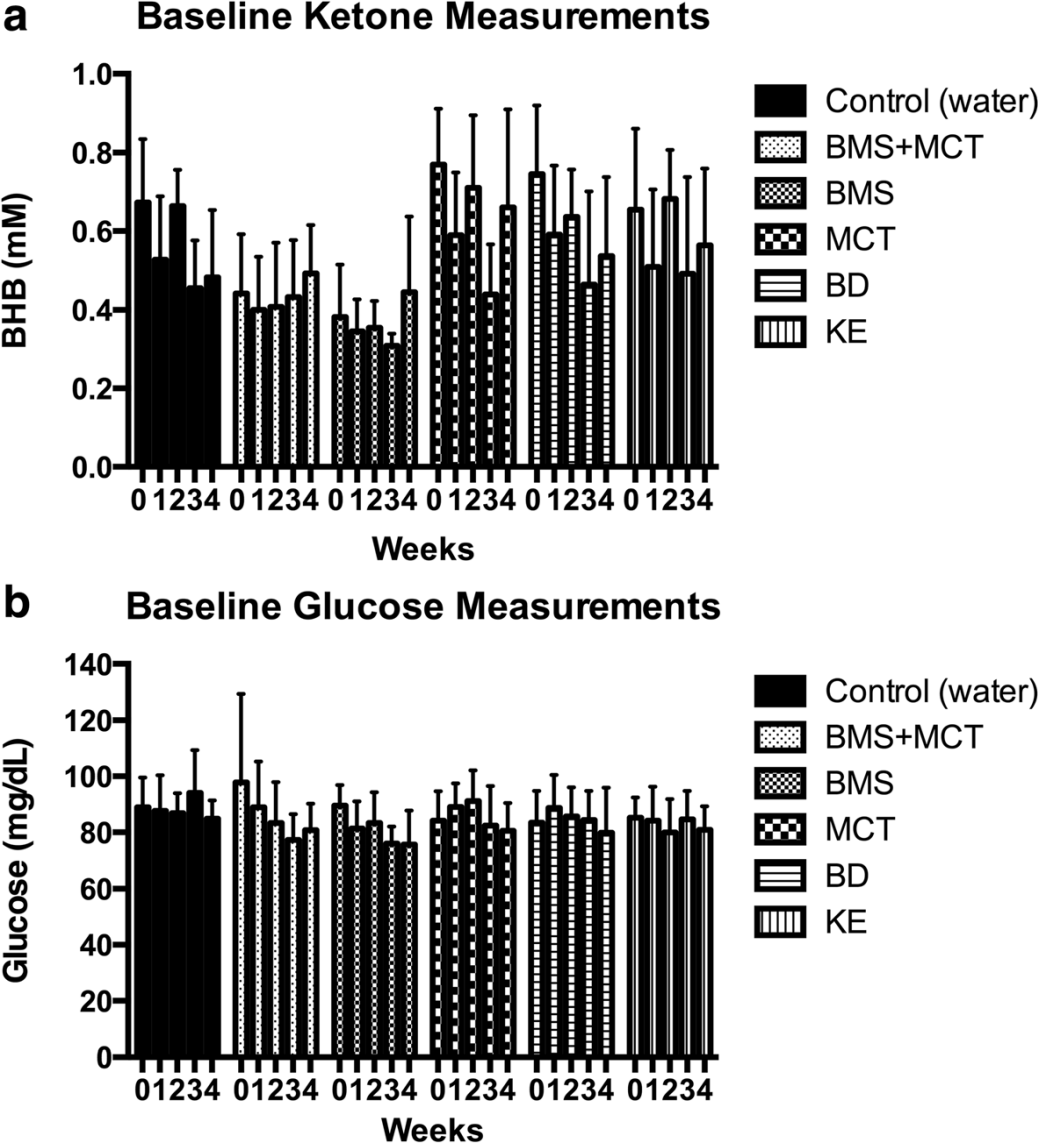
Effects of ketone supplementation on basal blood ketone and basal blood glucose levels: Rats administered ketone supplements did not have a significant change in basal blood ketone levels (**a**) or basal blood glucose levels (**b**) for the four week study. Two-Way ANOVA with Tukey’s post-hoc test, results considered significant if *p* < 0.05. Error bars represent mean (SD)

At day 29 of the study, animals were euthanized and brain, lungs, liver, kidneys, spleen and heart were harvested and weighed. Organ weights were normalized to body weight. Ketone supplementation did not significantly change brain, lung, kidney, or heart weights compared to controls (Fig. 5a, b, d, f). MCT supplemented animals had significantly larger livers compared to their body weight (*p* < 0.05) (Fig. 5c). Ketone supplements BMS + MCT, MCT and BD caused a significant reduction in spleen size (BMS + MCT *p* < 0.05, MCT *p* < 0.001, BD *p* < 0.05) (Fig. 5e). Rats administered KE gained significantly less weight over the entire study compared to controls. BMS + MCT, BMS, and BD supplemented rats gained significantly less weight than controls during weeks 2 – 4, and MCT animals gained less weight than controls at weeks 3 – 4 (Fig. 6). Increased gastric motility (increased bowel evacuation and changes to fecal consistency) was visually observed in rats supplemented with 10 g/kg MCT, most notably at the 8 and 12-h time points. All animals remained in healthy weight range for their age even though the rate of weight gain changed with ketone supplementation [53–54]. Food intake was not measured in this study. However, there was not a significant change in basal blood glucose or basal blood ketone levels over the 4 week study in any of the rats supplemented with ketones (Fig. 7).

## Discussion

Nutritional ketosis induced with the KD has proven effective for the metabolic management of seizures and potentially other disorders [1–26]. Here we present evidence that chronic administration of ketone supplements can induce a state of nutritional ketosis without the need for dietary carbohydrate restriction and with little or no effect on lipid biomarkers. The notion that we can produce the therapeutic effects of the KD with exogenous ketone supplementation is supported by our previous study which demonstrated that acutely administered KE supplementation delays central nervous system (CNS) oxygen toxicity seizures without the need for dietary restriction [29]. We propose that exogenous ketone supplementation could provide an alternative method of attaining the therapeutic benefits of nutritional ketosis, and as a means to further augment the therapeutic potential of the KD.

### Ketone supplementation causes little to no change in triglycerides and lipoproteins

One common concern regarding the KD is its purported potential to increase the risk of atherosclerosis by elevating blood cholesterol and triglyceride levels [55, 56]. This topic remains controversial as some, but not all, studies have demonstrated that the KD elevates blood levels of cholesterol and triglycerides [57–62]. Kwitervich and colleagues demonstrated an increase in low-density lipoprotein (LDL) and a decrease in high-density lipoprotein (HDL) in epileptic children fed the classical KD for two years [27]. In this study, total cholesterol increased by ~130 %, and stabilized at the elevated level over the 2-year period. A similar study demonstrated that the lipid profile returned to baseline in children who remained on the KD for six years [63]. Children typically remain on the diet for approximately two years then return to a diet of common fat and carbohydrate ingestion [64]. The implications of these findings are unclear, since the influence of cholesterol on cardiovascular health is controversial and macronutrient sources of the diet vary per study. In contrast to these studies, the majority of recent studies have suggested that the KD can actually lead to significant benefits in biomarkers of metabolic health, including blood lipid profiles [65–72]. In these studies, the KD positively altered blood lipids, decreasing total triglycerides and cholesterol while increasing the ratio of HDL to LDL [68–77]. Although, the KD is well-established in children, it has only recently been utilized as a strategy to control seizures in adults. In 2014, Schoeler and colleagues reported on the feasibility of the KD for adults, concluding that 39 % of individuals achieved > 50 % reduction in seizure frequency, similar to the results reported in pediatric studies. Patients experienced similar gastrointestinal adverse advents that have been previously described in pediatric patients, but they did not lead to discontinuation of the diet in any patient [78].

With oral ketone supplementation, we observed a significant elevation in blood βHB without dietary restriction and with little change in lipid biomarkers (Fig. 1). Over the 4 week study, MCT-supplemented rats demonstrated decreased HDL compared to controls. No significant changes were observed in any of the triglycerides or lipoproteins (HDL, LDL) with any of the remaining exogenously applied ketone supplements. It should be noted that the rats used for this study had not yet reached full adult body size [79]. Their normal growth rate and maturation was likely responsible for the changes in triglyceride and lipoprotein levels observed in the control animals over the 4 week study (baseline data not shown, no significant differences) [80, 81]. Future studies are needed to investigate the effect of ketone supplementation on fully mature and aged animals. Overall, our study suggests that oral ketone supplementation has little effect on the triglyceride or lipoprotein profile after 4 weeks. However, it is currently unknown if ketone supplementation would affect lipid biomarkers after a longer duration of consumption. Further studies are needed to determine the effects of ketone supplements on blood triglyceride and lipoproteins after chronic administration and as a means to further enhance the hyperketonemia and improve the lipid profile of the clinically implemented (4:1) KD.

LDL is the lipoprotein particle that is most often associated with atherosclerosis. LDL particles exist in different sizes: large molecules (Pattern A) or small molecules (Pattern B). Recent studies have investigated the importance of LDL-particle type and size rather than total concentration as being the source for cardiovascular risk [56]. Patients whose LDL particles are predominantly small and dense (Pattern B) have a greater risk of cardiovascular disease (CVD). It is thought that small, dense LDL particles are more able to penetrate the endothelium and cause in damage and inflammation [82–85]. Volek et al. reported that the KD increased the pattern and volume of LDL particles, which is considered to reduce cardiovascular risk [73]. Though we did not show a significant effect on LDL levels for ketone supplements, future chronic feeding studies will investigate the effects of ketone supplementation on lipidomic profile and LDL particle type and size.

### Therapeutic levels of hyperketonemia suppress blood glucose levels

We demonstrated that therapeutic ketosis could be induced without dietary (calorie or carbohydrate) restriction and that this acute elevation in blood ketones was significantly correlated with a reduction in blood glucose (Figs. 2, 3 and 4). The BMS ketone supplement did not significantly induce blood hyperketonemia or reduced glucose in the rats. The KE supplemented rats trended towards reduced glucose levels; however, the lower dose of this agent did not lower glucose significantly, as reported previously in acute response of mice [59]. MCTs have previously been shown to elicit a slight hypoglycemic effect by enhancing glucose utilization in both diabetic and non-diabetic patients [86–88]. Kashiwaya et al. demonstrated that both blood glucose and blood insulin decreased by approximately 50 % in rats fed a diet where 30 % of calories from starch were replaced with ketone esters for 14 days, suggesting that ketone supplementation increases insulin sensitivity or reduced hepatic glucose output [89]. This ketone-induced hypoglycemic effect has been previously reported in humans with IV infusions of ketone bodies [90, 91]. Recently, Mikkelsen et al. showed that a small increase in βHB concentration decreases glucose production by 14 % in post-absorptive health males [92]. However, this has not been previously reported with any of the oral exogenous ketone supplements we studied. Ketones are an efficient and sufficient energy substrate for the brain, and will therefore prevent side effects of hypoglycemia when blood levels are elevated and the patient is keto-adapted. This was most famously demonstrated by Owen et al. in 1967 wherein keto-adapted patients (starvation induced therapeutic ketosis) were given 20 IU of insulin. The blood glucose of fasted patients dropped to 1–2 mM, but they exhibited no hypoglycemic symptoms due to brain utilization of ketones for energy [93]. Therefore, ketones maintain brain metabolism and are neuroprotective during severe hypoglycemia. The rats in the MCT group had a correlation of blood ketone and glucose levels at week 4, whereas the combination of BMS + MCT produced a significant hypoglycemic correlation both at baseline and at week 4. No hypoglycemic symptoms were observed in the rats during this study. Insulin levels were not measured in this study; however, future ketone supplementation studies should measure the effects of exogenous ketones on insulin sensitivity with a glucose tolerance test. An increase in insulin sensitivity in combination with our observed hypoglycemic effect has potential therapy implications for glycemic control in T2D [40]. Furthermore, it should be noted that the KE metabolizes to both AcAc and βHB in 1:1 ratio [29]. The ketone monitor used in this study only measures βHB as levels of AcAc are more difficult to measure due to spontaneous decarboxylation to acetone; therefore, the total ketone levels (βHB + AcAc) measured were likely higher, specifically for the KE [14]. Interestingly, the 10 g/kg dose produced a delayed blood βHB peak for ketone supplements MCT and BMS + MCT. The higher dose of the ketogenic supplements elevated blood levels more substantially, and thus reached their maximum blood concentration later due to prolonged metabolic clearance. It must be noted that the dosage used in this study does not translate to human patients, since the metabolic physiology of rats is considerably higher. Future studies will be needed to determine optimal dosing for human patients.

### Effects of ketone supplementation on organ weight and body weight percentage

Ketone supplementation did not affect the size of the brain, lungs, kidneys or heart of rats. As previously mentioned, the rats were still growing during the experimental time frame; therefore, organ weights were normalized to body weight to determine if organ weight changed independently to growth. There could be several reasons why ketones influenced liver and spleen weight. The ratio of liver to body weight was significantly higher in the MCT supplemented animals (Fig. 5). MCTs are readily absorbed in the intestinal lumen and transported directly to the liver via hepatic portal circulation. When given a large bolus, such as in this study, the amount of MCTs in the liver will likely exceed the β-oxidation rate, causing the MCTs to be deposited in the liver as fat droplets [94]. The accumulated MCT droplets in the liver could explain the higher liver weight to body weight percentage observed with MCT supplemented rats. Future toxicology and histological studies will be needed to determine the cause of the observed hepatomegaly. It should be emphasized that the dose in this study is not optimized in humans. We speculate that an optimized human dose would be lower and may not cause hepatomegaly or potential fat accumulation. Nutritional ketosis achieved with the KD has been shown to decrease inflammatory markers such as TNF-α, IL-6, IL-8, MCP-1, E-selectin, I-CAM, and PAI-1 [8, 46], which may account for the observed decrease in spleen weight. As previously mentioned, Veech and colleagues demonstrated that exogenous supplementation of 5 mM βHB resulted in a 28 % increase in hydraulic work in the working perfused rat heart and a significant decrease in oxygen consumption [28, 41, 42]. Ketone bodies have been shown to increase cerebral blood flow and perfusion [95]. Also, ketone bodies have been shown to increase ATP synthesis and enhance the efficiency of ATP production [14, 28, 40]. It is possible that sustained ketosis results in enhanced cardiac efficiency and O_2_ consumption. Even though the size of the heart did not change for any of the ketone supplements, further analysis of tissues harvested from the ketone-supplemented rats will be needed to determine any morphological changes and to understand changes in organ size. It should be noted that the Harlan standard rodent chow 2018 is nutritionally complete and formulated with high-quality ingredients to optimize gestation, lactation, growth, and overall health of the animals. The same cannot be said for the standard American diet (SAD). Therefore, we plan to investigate the effects of ketone supplements administered with the SAD to determine if similar effects will be seen when the micronutrient deficiencies and macronutrient profile mimics what most Americans consume.

MCT oil has recently been used to induce nutritional ketosis although it produces dose-dependent gastrointestinal (GI) side effects in humans that limit the potential for its use to significantly elevate ketones (>0.5 mM). Despite these limitations, Azzam and colleagues published a case report in which a 43-year-old-man had a significant decrease in seizure frequency after supplementing his diet with 4 tablespoons of MCT oil twice daily [96]. An attempt to increase his dosage to 5 tablespoons twice daily was halted by severe GI intolerance. Henderson et al. observed that 20 % of patients reported GI side effects with a 20 g dose of ketogenic agent AC-1202 in a double blind trial in mild to moderate Alzheimer’s patients [24]. We visually observed similar gastrointestinal side effects (loose stools) in the rats treated with MCT oil in our study. Rats were closely monitored to avoid dehydration, and gastric motility returned to normal between 12–24 h. Interestingly, the BMS + MCT supplement elevated βHB similarly to MCT oil alone, without causing the adverse gastrointestinal effects seen in MCT-supplemented rats. However, this could be due to the fact in a 10 g/kg dose of BMS + MCT, only 5 g/kg is MCT alone, which is less than the 10 g/kg dose that elicits the GI side effects. This suggests that this novel combination may provide a more useful therapeutic option than MCT oil alone, which is limited in its ability to elevate ketones in humans.

Exogenously delivered ketone supplements significantly altered rat weight gain for the duration of the study (Fig. 6). However, rats did not lose weight and maintained a healthy range for their age. Rats have been shown to effectively balance their caloric intake to prevent weight loss/gain [97–99]. Due to the caloric density of the exogenous ketone supplements (Table 1) it is possible for the rats to eat less of the standard rodent chow and therefore less carbohydrates while maintaining their caloric intake. Food intake was not measured for this study. However, if there was a significant carbohydrate restriction there would be a signifcant change in basal blood ketone and blood glucose levels. As the hallmark to the KD, carbohydrate restriction increases blood ketone levels and reduces blood glucose levels. Neither an increase in basal blood ketone levels nor a decrease in basal blood glucose levels was observed in this study (Fig. 7). Additionally, if there were an overall blood glucose decrease due to a change in food intake, this would not explain the rapid reduction (within 30 min) in blood glucose correlated with an elevation of blood ketone levels after an intragastric bolus of ketone supplement (Figs. 2, 3 and 4).

## Conclusions

Several studies have investigated the safety and efficacy of ketone supplements for disease states such as AD and Parkinson’s disease, and well as for parenteral nutrition [40, 48–50, 100–103]. Our research demonstrates that several forms of dietary ketone supplementation can effectively elevate blood ketone levels and achieve deleted: therapeutic nutritional ketosis without the need for dietary carbohydrate restriction. We also demonstrated that ketosis achieved with exogenous ketone supplementation can reduce blood glucose, and this is inversely associated with the blood ketone levels. Although preliminary results are encouraging, further studies are needed to determine if oral ketone supplementation can produce the same therapeutic benefits as the classic KD in the broad-spectrum of KD-responsive disease states . Additionally, further experiments need to be conducted to see if the exogenous ketone supplementation affects the same physiological features as the KD (i.e. ROS, inflammation, ATP production). Ketone supplementation could be used as an alternative method for inducing ketosis in patients uninterested in attempting the KD or those who have previously had difficulty implementing the KD because of palatability issues, gall bladder removal, liver abnormalities, or intolerance to fat. Additional experiments should be conducted to see if ketone supplementation could be used in conjunction with the KD to assist and ease the transition to nutrition ketosis and enhance the speed of keto-adaptation. In this study we have demonstrated the ability of several ketone supplements to elevate blood ketone levels, providing multiple options to induce therapeutic ketosis based on patient need. Though additional studies are needed to determine the therapeutic potential of ketone supplementation, many patients that previously were unable to benefit from the KD may now have an alternate method of achieving therapeutic ketosis. Ketone supplementation may also represent a means to further augment ketonemia in those responsive to therapeutic ketosis, especially in those individuals where maintaining low glucose is important.

## Abbreviations

AcAc: acetoacetate
ALS: amyotrophic lateral sclerosis
AD: Alzheimer’s
βHB: beta-hydroxybutyrate
BMS: sodium/potassium βHB mineral salt
BMS + MCT: BMS + MCT 1:1 mixture
BD: 1,3-butanediol
CNS: central nervous system
HDL: high density lipoprotein
HDACs: histone deacetylases
LDL: low density lipoprotein
KD: ketogenic diet
KE: 1, 3-butanediol acetoacetate diester/ketone ester
MCT: medium chain triglyceride oil
PCOS: polycystic ovary syndrome
ROS: reactive oxygen species
SAD: standard American diet
TBI: traumatic brain injury
T2D: type-2 diabetes
